# Finding New Fundamental Pieces for the Bacterial Cell Division Puzzle

**DOI:** 10.1128/mbio.00737-22

**Published:** 2022-06-23

**Authors:** Dorte Frees, Hanne Ingmer

**Affiliations:** a Department of Veterinary and Animal Sciences, Faculty of Health and Medical Sciences, University of Copenhagen, Copenhagen, Denmark

**Keywords:** CRISPRi, essential genes, MRSA, PBP1, *Staphylococcus aureus*, cell division

## Abstract

The division of bacterial cells into two daughter cells requires a precise balance of more than a dozen highly conserved proteins that coordinate chromosome segregation with the synthesis of the novel cell envelope. The paradigms of cell division were established in rod-shaped bacteria and this fundamental process is far less characterized in spherical bacteria. In a search for novel, essential cell division proteins in Staphylococci, Myrbråten et al. used combined depletion and subcellular localization analyses to identify the staphylococcal morphology determinant, SmdA, that is exclusively found in cocci. Knockdown of *smdA* results in severe division defects and increased sensitivity to cell wall targeting antibiotics. Although determining the precise role of SmdA in S. aureus cell division will require further research, this study provides a striking example of how researchers can assign functions to genes that are too fundamental to cell biology to allow genetic inactivation.

## COMMENTARY

A central goal in biology is to delineate the molecular pathways that allow cells to grow and divide. While genome sequencing has become fast and easy, assigning functions to newly identified genes remains challenging and bacterial genomes encode hundreds of hypothetical proteins with functions yet to be assigned. Protein function is traditionally assigned based on mutant phenotypes, but this approach is hampered if the gene is essential or if deletion of the gene confers a severe fitness cost. In addition, essential genes are missing from the mutant libraries that have otherwise revolutionized genome-wide searches for genetic loci supporting growth in diverse conditions. Accordingly, essential genes remain relatively understudied despite being involved in the most fundamental processes of life. The toolbox is, however, expanding, and methods such as CRISPR interference (CRISPRi) now offer an efficient approach for knockdown phenotypic analysis of essential genes (1). In the CRISPRi approach, a single guide RNA, containing a gene-specific base-pairing region and a structured region for interaction with deactivated Cas9, is deployed to sterically block the transcription of specific genes (1).

For bacterial pathogens, characterization of essential genes holds promise to identify novel targets for antibiotic development. In a recent study, Myrbråten et al. used CRISPRi gene knockdown to identify a novel factor that is fundamental for proper cell division in the opportunistic human pathogen, Staphylococcus aureus (2). Treatment of S. aureus infections is hampered by the worldwide dissemination of methicillin-resistant S. aureus (MRSA) that are resistant to penicillin and almost all other β-lactam antibiotics (3). In MRSA strains, β-lactam resistance is provided by PBP2a, a penicillin-binding protein (PBP) that cross-links the peptidoglycan (PG) even in the presence of β-lactams, which inhibits the function of native PBPs (3). Essential genes impacting cell division are particularly attractive targets for antibiotic development as MRSA strains can be resensitized to the superior β-lactam antibiotics by hindering the function of central cell division proteins (4).

**FIG 1:**
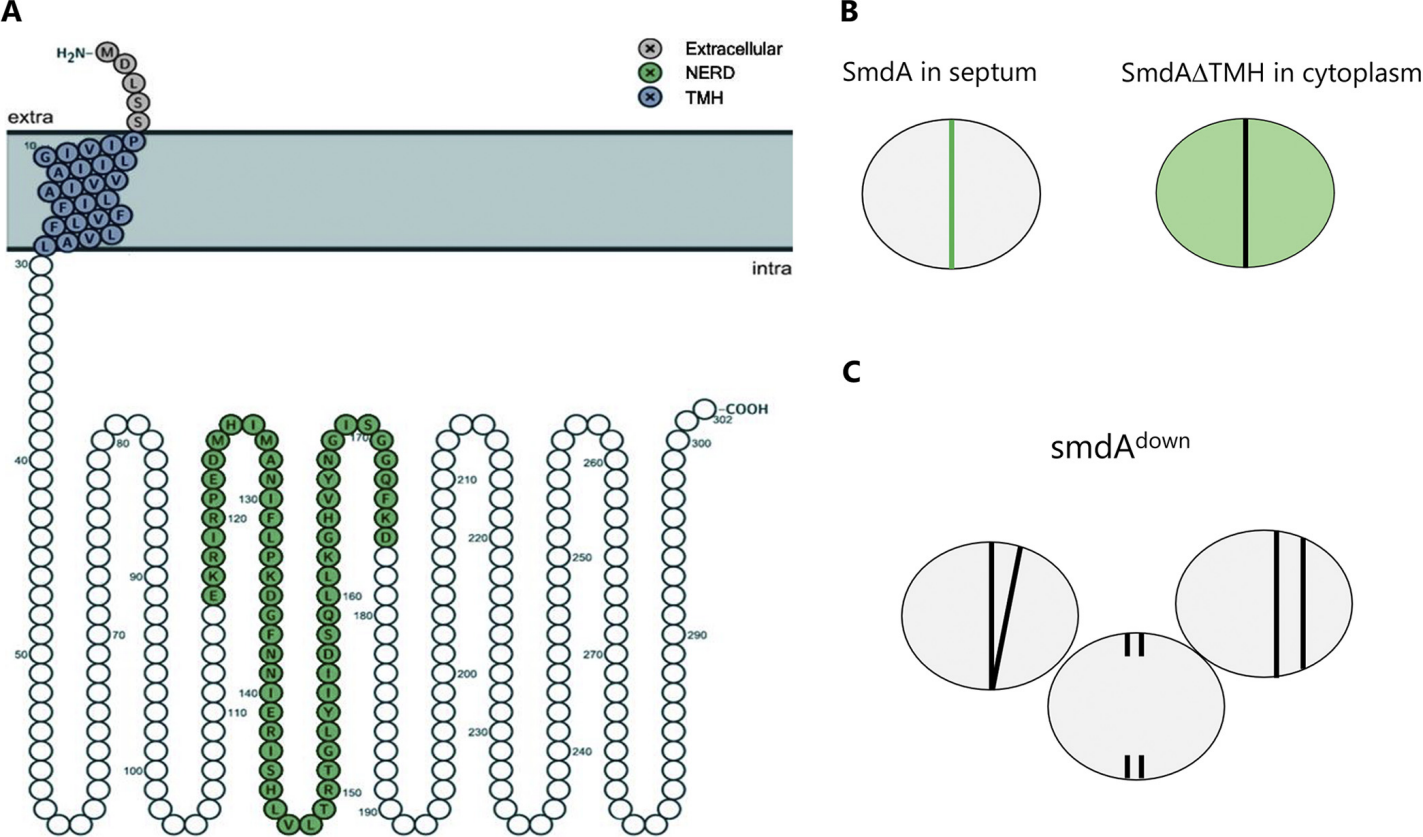
Identification of staphylococcal morphology determinant, SmdA. (A) The predicted topology of SmdA shows membrane anchoring via a single N-terminal transmembrane helix (TMH) and a conserved NERD domain in the cytoplasmic part of the protein. (B) SmdA has a septum-enriched localization that depends on the N-terminal TMH. (C) CRISPRi knockdown of *smdA* resulted in division defects and aberrant septum formation. See text for details.

In S. aureus, approximately three hundred genes are predicted to be essential (5–7). To identify those with a role in cell division, Myrbråten et al. focused their attention on predicted essential staphylococcal proteins with no annotated functions and performed combined subcellular localization and CRISPRi knockdown analyses to identify proteins with a septal localization that, if depleted, resulted in aberrant cell division. This screening approach led to the identification of staphylococcal morphology determinant, SmdA, a membrane-attached protein (Fig. 1A) which is fully conserved in species within the staphylococcal family and other cocci but is absent from rod-shaped bacteria. SmdA attaches to the membrane through a single N-terminal transmembrane spanning helix and deletion of this helix displaced SmdA from the septal site (Fig. 1B). CRISPRi-depletion of SmdA resulted in cells with multiple aberrant septa (Fig. 1C) that formed clusters. Overexpression of SmdA resulted in similar phenotypes illustrating that crucial processes such as septum placement and autolytic splitting of daughter cells are dependent on proper SmdA levels. The SmdA depleted cells were, however, able to form colonies indicating that SmdA, rather than being essential for growth, confers a severe growth defect. Strikingly, depletion of SmdA increased S. aureus sensitivity to β-lactam antibiotics, especially, in the highly resistant MRSA strain, COL.

The cell wall of Gram-positive bacteria consists of a thick layer of peptidoglycan (PG) decorated with teichoic acids (TA) that are glycopolymers either anchored to the cell wall (wall teichoic acids, WTA) or embedded in the cell membrane (lipoteichoic acids, LTA) (8). Interestingly, inhibitors of TA biosynthesis sensitize MRSA strains to β-lactams revealing a fundamental link between TA and PG synthesis (9, 10). In S. aureus, WTA and LTA appear to overlap in function as either one of these structures is required for cell viability (11). Both LTAs and WTAs have yet unidentified roles in cell division with mutants defective in either LTA or WTA synthesis, likewise to SmdA depleted cells, displaying impaired cell separation and multiple, aberrantly placed, division septa (11, 12). Moreover, as depletion of SmdA increased the susceptibility of S. aureus to inhibitors of WTA and LTA biosynthesis, this raised the possibility that SmdA contributes to S. aureus cell division via impacting TA biosynthesis. Depletion of SmdA did, however, not have any visible effect on TA biosynthesis. Thus, rather than impacting the expression or composition of TAs, SmdA and TAs may have overlapping roles in septum placement and/or separation of daughter cells. Of note, we recently made related findings for the auxiliary factor, AuxA that, like SmdA, is a membrane protein restricted to and conserved among coccoid bacteria, whose inactivation renders MRSA susceptible to β-lactam and inhibitors of the TA synthesis pathway (13). Further characterization of cocci-restricted cell division proteins like SmdA and AuxA will likely lead to novel insights into the mechanisms that determine the spherical morphology.

Pulldown experiments and bacterial two-hybrid assays suggested direct interaction between SmdA and core factors of the divisome, including the early division protein, EzrA, and the penicillin-binding proteins PBP1, PBP2, and PBP3, indicating that SmdA may control septal PG synthesis by protein-protein interaction. Despite coccoid bacteria not possessing the classical machinery that allows cell elongation, the MreB-based elongasome, S. aureus was recently shown to be capable of slight elongation (14). In S. aureus, the lateral PG incorporation at the midcell is accomplished by the transglycosylase, RodA, and the transpeptidases of PBP3, while the FtsW-PBP1 pair contributes to inward PG synthesis at the septal site (14). Additionally, PBP1 was proposed to stabilize the divisome at the midcell and to take part in a checkpoint-type mechanism that coordinates autolytic splitting of daughter cells with septum synthesis (14, 15). Notably, SmdA also seems to directly interact with Atl, a key autolysin in cell division. Hence, SmdA could together with PBP1 be involved in coordinating septal PG synthesis and degradation. SmdA localizes to the septal site after the early divisome proteins, and the transmembrane helix is required for this localization. Strikingly, overexpression of SmdA without the membrane-spanning helix resulted in a more extreme phenotype than overexpression of full-length SmdA. The C-terminal cytoplasmic part of SmdA has partial homology to a so-called nuclease-related domain (NERD in Fig. 1A). Myrbråten et al., for the first time, identify residues that are critical for the function of this enigmatic domain that despite its conservation in bacterial, archaeal, and plant proteins remains functionally uncharacterized.

In all living cells, molecular chaperones facilitate protein folding, unfolding, and interaction. Strikingly, the pulldown experiment additionally suggested interactions between SmdA and several proteins involved in protein folding, including the cytoplasmic chaperones ClpB, ClpC, and the extracellular PrsA foldase. Cell division is an exceptionally complicated cycle of events that requires strict spatiotemporal control of assembly, remodeling, and disassembly of the divisome complex (16). Presently, the contribution of molecular chaperones to divisome assembly and rearrangement remains largely unexplored, but recent studies have shed light on the important contributions of chaperones to the divisomal machinery. As an example, the major resistance determinant in MRSA, the PBP2a transpeptidase is prone to misfolding and needs assistance from the membrane-bound PrsA chaperone to fold correctly (17). Accordingly, the PrsA foldase protein is a modulator of β-lactam resistance. Also, we have shown that the ClpX chaperone plays a temperature-dependent role in staphylococcal septum synthesis, resulting in severe morphological changes and lysis of S. aureus *clpX* cells at 30°C but not at 37°C (18). The ClpX unfoldase is best characterized for its ability to select and unfold substrates for degradation by the ClpP protease, but the role of ClpX in S. aureus septum synthesis is independent of ClpP. We envision that the unfoldase activity of ClpX becomes crucial for assisting rearrangements in the divisome that can proceed spontaneously at optimal temperatures but become rate-limiting as the temperature decreases. The putative association between SmdA and ClpC and ClpB is indicative of a functional link between cell division and these chaperones that are mostly known for their role during heat stress and other conditions resulting in proteotoxic stress.
